# Repetitive transcranial magnetic stimulation for the treatment of suicidality in opioid use disorder: a pilot feasibility randomized controlled trial

**DOI:** 10.1192/j.eurpsy.2025.28

**Published:** 2025-03-05

**Authors:** Victor M. Tang, Bernard Le Foll, Zafiris J. Daskalakis, An-Li Wang, Leslie Buckley, Daniel M. Blumberger, Daphne Voineskos

**Affiliations:** 1 Institute of Medical Science, University of Toronto, Toronto, ON, Canada; 2 Department of Psychiatry, Temerty Faculty of Medicine, University of Toronto, Toronto, ON, Canada; 3 Institute for Mental Health Policy Research, Centre for Addiction and Mental Health, Toronto, ON, Canada; 4 Addictions Division, Centre for Addiction and Mental Health, Toronto, ON, Canada; 5 Campbell Family Mental Health Research Institute, Centre for Addiction and Mental Health, Toronto, ON, Canada; 6 Translational Addiction Research Laboratory, Centre for Addiction and Mental Health, Toronto, ON, Canada; 7 Department of Pharmacology and Toxicology, Faculty of Medicine, University of Toronto, Toronto, ON, Canada; 8 Department of Family and Community Medicine, University of Toronto, Toronto, ON, Canada; 9 Waypoint Research Institute, Waypoint Centre for Mental Health Care, Penetanguishene, ON Canada; 10 Department of Psychiatry, University of California, San Diego, CA, USA; 11 Icahn School of Medicine at Mount Sinai, New York, NY, USA; 12 Temerty Centre for Therapeutic Brain Intervention, Centre for Addiction and Mental Health, Toronto, ON, Canada; 13 Poul Hansen Family Centre for Depression, Krembil Research Institute, Toronto Western Hospital, University Health Network, Toronto, ON, Canada

**Keywords:** major depressive disorder, opioid use disorder, suicide, theta burst stimulation, transcranial magnetic stimulation

## Abstract

**Background:**

Opioid use disorder (OUD) is a devastating condition with frequent suicidality, contributing to overdose deaths. Theta burst stimulation (TBS) to the dorsolateral prefrontal cortex (DLPFC) is used to treat major depressive disorder (MDD) and is effective in treating suicidal ideation. We piloted a randomized, double-blind, sham-controlled trial of bilateral rTMS for patients with OUD and MDD experiencing suicidality.

**Methods:**

Sequential bilateral TBS was delivered guided by structural neuroimaging: continuous TBS to the right then intermittent TBS to the left DLPFC, daily (20 treatments). The primary objective was to determine the feasibility of this population. The primary clinical outcome was the scale for suicidal ideation (SSI), secondary outcomes included depressive symptoms and opioid cue-induced craving. ClinicalTrials.gov: NCT04785456.

**Results:**

Eighty-seven individuals were pre-screened. The most common reasons for ineligibility included being unreachable by the study team, difficulty with scheduling/travel requirements, and medical/psychiatric instability. Six participants (5:1 M:F) were enrolled (3/arm), four had a fentanyl use history; two completed per protocol (1/arm). Of the participants with follow-up data, SSI scores decreased in 2/3 in the sham arm and 2/2 in the active arm; depression and opioid craving scores decreased in all participants.

**Conclusion:**

We present the first data piloting a structural neuroimaging-guided, multi-session rTMS treatment course in outpatients with suicidality and OUD in the current North American context. Recruitment and retention were the main challenges given the highly unstable medical and psychosocial context of this patient population. Future trials should consider a suitable environment to improve the feasibility of delivering this treatment.

## Introduction

Opioid misuse is a prevalent issue, in North America now referred to as an opioid epidemic, and has been worsening and continues to escalate [1]. An estimated 6-7 million individuals meet criteria for Opioid Use Disorder (OUD) in the United States [2], and is associated with high morbidity, mortality, and service utilization [3]. Recently, changes in opioid drug supply have resulted in a tenfold increase in opioid-related deaths, due in large part to access to synthetic opioids [1], increasing by over 15% in one year [4]. Qualitative studies suggest that many who survive opioid overdoses reported a desire to have died [5]. In people with any mental illness, a comorbid substance use disorder increases the risk of completed suicide threefold [6]. Importantly, suicidality may be an overlooked contributor to OUD-related mortality [7]. Comorbidity with major depressive disorder (MDD) is common in OUD [8], and appears to confer an increased risk of suicide beyond that of each disorder alone [9, 10]. Specifically addressing suicidality in people with OUD and comorbid MDD may be a promising avenue to decrease mortality rates.

Repetitive transcranial magnetic stimulation (rTMS) to the dorsolateral prefrontal cortex (DLPFC) is a form of non-invasive neuromodulation used therapeutically in MDD resistant to pharmacotherapy or psychotherapy [11]. In addition to treating MDD, rTMS has demonstrated efficacy in decreasing suicidal ideation [12, 13]. In a secondary analysis of two randomized controlled trials, the rTMS protocol that appeared most efficacious for reducing suicidal ideation was a bilateral approach with inhibitory stimulation to the right DLPFC and excitatory stimulation to the left DLPFC, perhaps due in part to the DLPFC exerting executive and cognitive control of negative emotion [14]. rTMS is also being increasingly explored as a treatment option for OUD, with some early clinical trials demonstrating a reduction of opioid craving [15]. In studies of rTMS targeting OUD specifically, there has been evidence that co-occurring symptoms of depression and anxiety have improved with treatment [16]. Furthermore, the use of rTMS in treating patients with complex mental illness and substance use comorbidity appears to be feasible but requires greater study given its prevalence and the clinical need for more therapeutic options [16].

The development of an effective intervention for suicidality in patients with comorbid OUD and MDD remains a major therapeutic challenge for clinicians and healthcare systems. Evidence suggests rTMS is effective in treating MDD and suicidality and in reducing opioid cravings; thus, it is well-positioned to treat those who have these conditions concurrently. The objective of the presented study was to evaluate the feasibility of rTMS in treating suicidality in patients with co-occurring OUD and MDD in a randomized, sham-controlled trial. We designed an approach bilaterally targeting the DLPFC with theta-burst stimulation (TBS), which delivers similar antidepressant effects as standard rTMS over a shortened treatment duration time [17].

## Methods

### Study design

The research protocol was approved by the research ethics board and conducted at the Centre for Addiction and Mental Health in Toronto, Ontario, Canada. A randomized, double-blind, sham-controlled pilot trial of bilateral theta burst stimulation (TBS) in a 1:1 ratio for participants with opioid use disorder (OUD) experiencing suicidality in the context of a major depressive episode. All participants received active or sham treatment to the DLPFC bilaterally 5 days per week for 4 weeks. Stimulation was delivered with continuous TBS (cTBS) to the right DLPFC followed by intermittent TBS (iTBS) to the left DLPFC. Anatomic targeting of the DLPFC (below) was determined through individual Magnetic Resonance Imaging (MRI) using the Brainsight neuronavigation system. Participants were assessed clinically at baseline, weekly, at the end of treatment (i.e., week 4) and 4 weeks post-treatment (i.e., week 8), for changes in suicidality, MDD symptoms, and opioid cravings. The trial was designed according to international CONSORT guidelines [18] and was registered in an international clinical trial registry (clinicaltrials.gov NCT04785456).

### Participants

Individuals were included if: (1) capable of providing informed consent, (2) aged 18–60 years, (3) they met the criteria for Mini-International Neuropsychiatric Interview (MINI) version 5 confirmed DSM-IV diagnoses of Opioid Dependence (or if opioid dependence is in remission, is still being treated with evidence-based medication for opioid use disorder) AND MDD, (4) on a stable treatment regimen without any change in antidepressant medications or dosages in the previous 30 days and opioid agonist therapy in the previous 7 days, (5) met baseline scores of ≥4 on the scale for suicidal ideation (SSI). Exclusion criteria included: (1) pregnancy, (2) diagnosis of bipolar disorder, psychotic disorder, or experiencing any current psychotic symptoms, (3) exposure to previous rTMS, (4) known active seizure disorder, (5) significant head injury with an imaging verified lesion, (6) unstable medical illness, (7) presence of cardiac pacemaker, intracranial implant, or metal in the cranium, (8) >2 mg lorazepam (or other benzodiazepine at an equivalent dose) or any anticonvulsant medication.

Other safety considerations and withdrawal criteria included: (1) treatment for the day was not delivered if the participant presented for any study visit while intoxicated or in active withdrawal, (2) participants were withdrawn from the study if substance use became unstable or escalated in a way that could increase seizure risk or impact safety while receiving rTMS.

### Intervention

Localization to DLPFC was performed using neuronavigation with the Brainsight (Rogue Research, version 2.5.1), system using T1 weighted MRI scans acquired on the CAMH 3T scanner obtained with 7 fiducial markers in place. Stimulation was directed similarly in each hemisphere: at the junction of the middle and anterior 1/3rd of the middle frontal gyrus (Talairach co-ordinates (x,y,z) = −38, 44, 26 for the left hemisphere and 38, 44, 26 for the right) corresponding with posterior regions of BA9 which overlap with the superior section of BA46 [14]. TBS was administered using the MagPro X100 device equipped with a Cool-B70 A/P coil and Qooler fluid-cooling device (MagVenture, Farum, Denmark) positioned under MRI guidance using Brainsight. The Active-Placebo (A/P) B70 coil has one coil for active stimulation and the coil on the opposite side for sham stimulation, with an electronic sensor that records coil orientation. Bilateral TBS was delivered in the following pulse train parameters that have been previously established [17, 19]: first, continuous TBS (cTBS) over the R-DLPFC as 40s uninterrupted bursts (600 pulses), then intermittent TBS (iTBS) over the L-DLPFC: triplet 50Hz bursts, repeated at 5 Hz, 2 s on, and 8s off, (600 pulses per session, total duration of 3 min 9 s). Stimulation intensity was titrated up to 120% resting motor threshold (RMT) over the first several sessions.

### Assessments

Assessment of primary clinical outcomes was conducted at baseline and at the end of each of the four weeks of TBS. After the completion of TBS, there was a follow-up assessment 4 weeks after the treatment course had ended. The primary clinical outcome was suicidality assessed using the Scale for Suicidal Ideation (SSI) [20, 21]. Remission of suicidality was defined as SSI < 4 and ≥ 50% decrease from baseline over 2 consecutive assessments. Depressive symptoms were assessed using the 17-item Hamilton Rating Scale for Depression (HRSD-17) [17, 22]. Remission of the current depressive episode was defined as HRSD-17 score ≤ 7 and ≥ 60% decrease from baseline over 2 consecutive ratings. Opioid craving was assessed according to previously published methods [23, 24]. Participants were asked to rate their cravings on a visual analogue scale from 0 to 100 (0 = no cravings, 100 = very likely to use) before and after being exposed to a video of opioid use. The duration of the visual cue was 2 minutes with the content matched to the participant’s preferred route of administration (i.e., injection, inhalation, or ingestion of opioids) to maximize craving induction. The cue-induced craving score was computed as the numerical difference between the post-cue craving score minus the pre-cue craving score for each participant. Lastly, substance use was determined using the Timeline Followback method [25] and urine toxicology drug testing with a qualitative one-step immunoassay rapid test done on-site.

### Randomization and blinding

Treatment technicians, participants, and raters were all blinded to group allocation. For technician blinding, the patient’s study ID was entered into the device and a software-controlled switch automatically selected the active or sham coil for stimulation according to randomization, with the same coil type selected for each session. The sham coil generated auditory and somatosensory (vibratory) stimuli identical to the active stimulation. These methods have been previously shown to be effective at blinding participants and technicians at our centre [26]. Blinding was also supported by having participants without any previous history of having rTMS treatment, and our research assistants used a standardized script explaining randomization without reference to rTMS treatment. Randomization was using a permuted block method with a random number generator. Study personnel were blinded to randomization block sizes. Randomization of participants was managed by a research assistant external to the study.

## Results

**Figure 1.**
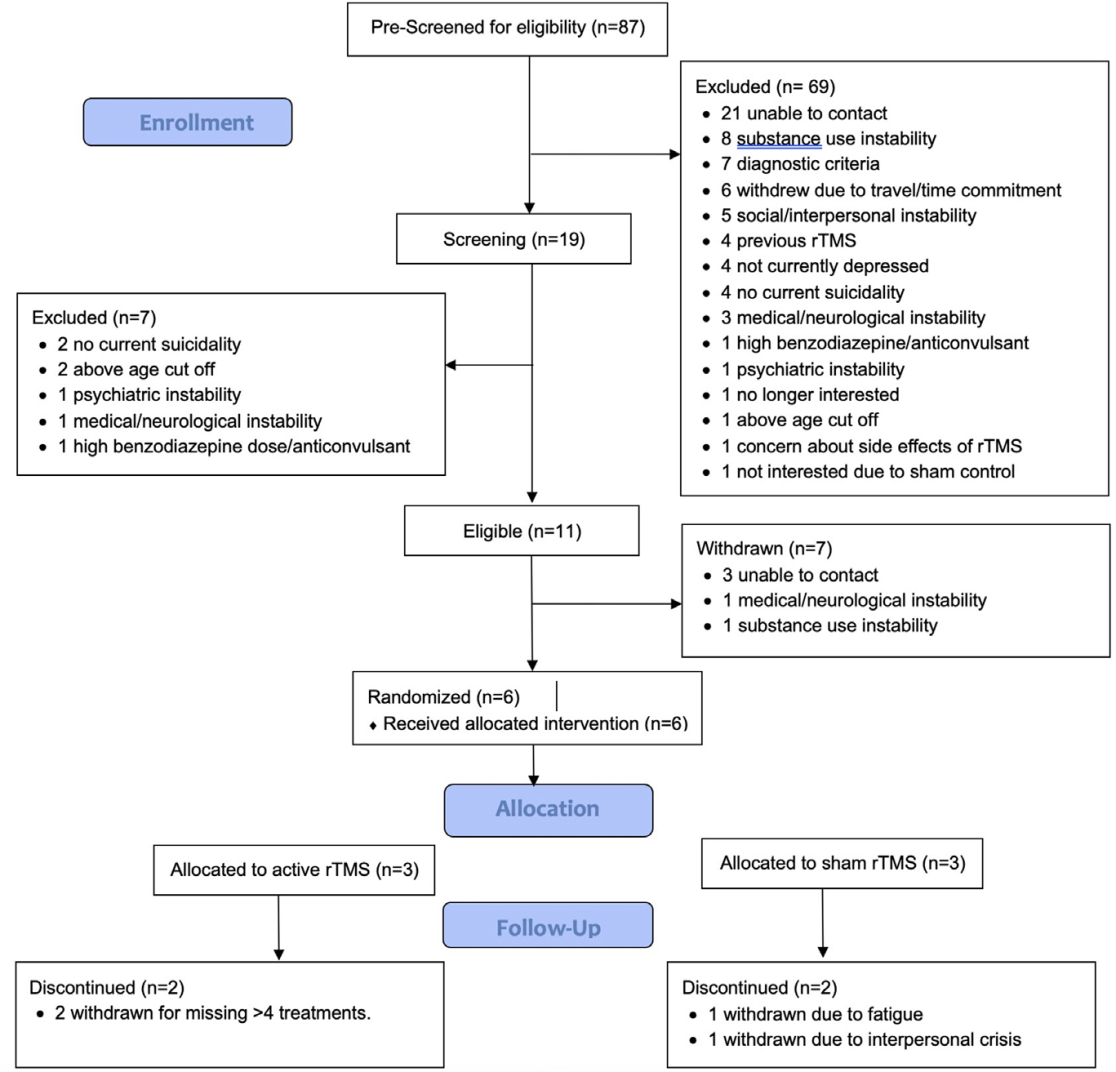
CONSORT flow diagram for patient enrollment and dropouts.

From August 2021 to September 2023, 87 participants were referred to the study. In total, 19 were screened, 11 were eligible and enrolled, 6 randomized to either rTMS or sham, and 2 completed the study per protocol. Participant flow and reasons for drop out/withdrawal are outlined in Figure 1. Owing to difficulties with recruitment and retention with the length of time of the study, recruitment was stopped in December 2023. Therefore, the trial protocol was determined to be infeasible with respect to recruitment and retention. However, other aspects of the trial protocol were determined to be feasible, including randomization, use of the sham-control procedures, administration of the MRI-guided, bilateral TBS protocol to this patient population, and suicidality, depression, and substance use measures.

For the six randomized participants, three were randomized to sham and three to active rTMS. One participant per arm completed the trial per protocol, with a range of 1-20 treatments completed (M = 11.8, SD = 7.4). Two were withdrawn due to exceeding the number of allowable missed treatments (without adequate notice or reason). One was withdrawn due to a severe interpersonal crisis and one was withdrawn due to nonspecific pre-existing chronic insomnia that was exacerbated by the study schedule. There were 5 males and 1 female, with age ranges from 25 to 55y (40.2y ± 10.9). All participants met the criteria for MDD, with baseline HRSD-17 ranging from 12 to 32 (M = 22.3, SD = 6.8) where a cut-off of ≥17 indicated moderate depressive severity. Baseline suicidality according to the SSI ranged from 8 to 19, M = 11.7, SD = 4.7. Demographic and clinical details may be found in Table 1.

**Table 1.** Participant demographics and baseline characteristics

Participant	Age	Sex	Race/Ethnicity	Housing	Comorbidity	Baseline SSI	Baseline HRSD-17	Opioid history	Medications
1	36	M	White – North American	Home rented by family and occasionally experiencing homelessness	PTSD, agoraphobia, GAD	10	12	Injection fentanyl	Buprenorphine/naloxone oral 4 mg and extended release subcutaneous 300 mg monthly, duloxetine 90 mg
2	40	M	White – North American	Home owned by family	Panic disorder, GAD, tinnitus, carpal tunnel	8	32	Insufflation/smoking fentanyl	Methadone 100 mg, venlafaxine 15 0 mg, mirtazapine 45 mg, trazodone 15 0 mg
3	25	M	Middle Eastern	Home rented by family	Agoraphobia, social phobia, GAD, HIV	16	18	Injection fentanyl	Buprenorphine extended release subcutaneous 300 mg mon, vortioxetine 10 mg, mirtazapine 45 mg, Bictegravir/emtricitabine/tenofovir alafenamide 50 mg/200 mg/25 mg
4	55	M	White – North American	Rental room	Neuropathic pain, tinnitus, BPH	8	25	Injection oxycodone	Buprenorphine/naloxone oral 24 mg, duloxetine 60 mg, tamsuosin 0.4 mg, furosemide 40 mg, melatonin 20 mg
5	50	M	East Asian	Rental apartment	Sleep apnea, hepatic steatosis, hypercholesterolemia, fibromyalgia	9	24	Oral oxycodone	Buprenorphine/naloxone 2.5 mg, venlafaxine50mg, pantoprazole 40 mg, fenofibrate 145 mg twice daily, rosuvastatin 10 mg, ciclesonide 50 mcg, salbutamol 100 mcg
6	35	F	White-European	Transitional supportive housing	PTSD, agoraphobia, GAD	19	23	Smoking fentanyl	Buprenorphine extended release subcutaneous 300 mg monthly

Abbreviations: BPH, benign prostatic hyperplasia; GAD, generalized anxiety disorder; HIV, human immunodeficiency virus positive; PTSD, posttraumatic stress disorder.

### Clinical outcomes

**Figure 2.**
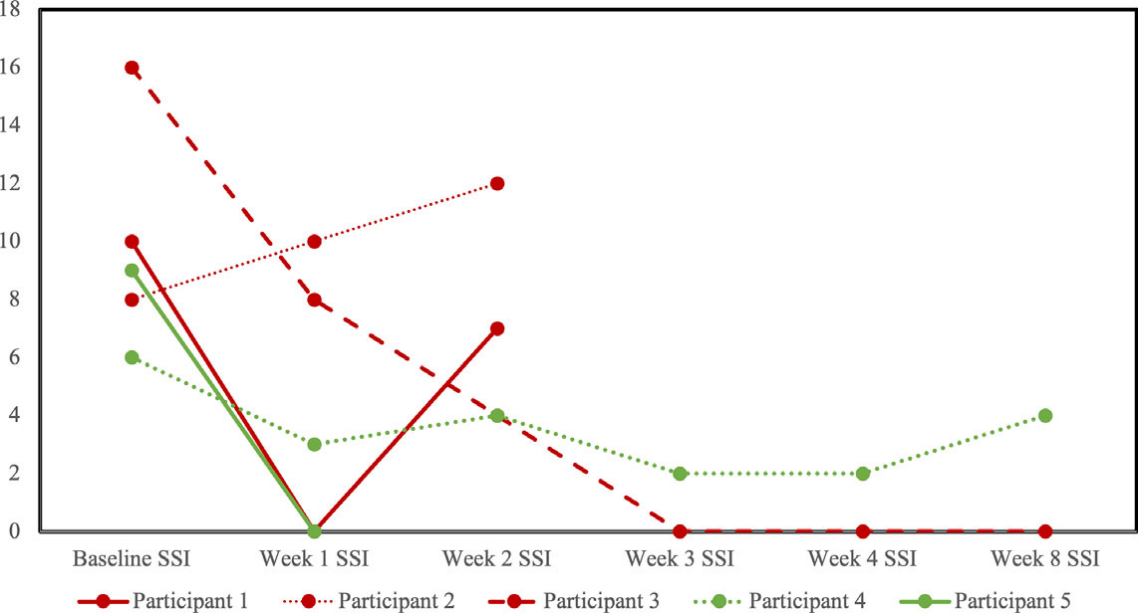
Profile plot of individual participants receiving sham (red) or active (green) repetitive transcranial magnetic stimulation (rTMS) and changes in the scale for suicidal ideation (SSI).

Five participants completed at least one follow-up visit, allowing for the assessment of change in clinical scores: three in the sham group and two in the active rTMS group. The two participants in the active rTMS arm reported decreases in SSI from baseline to last observation (Figure 2). The one per protocol completer reached remission criteria by the end of rTMS (SSI = 2), with improvements maintained at the 4 week follow visit, just shy of remission criteria (SSI = 4), In the sham group, SSI score decreased in two out of the three participants from baseline to the last observation, and the one per protocol completer reached remission criteria that was maintained at 4 week follow up (SSI = 0).

**Figure 3.**
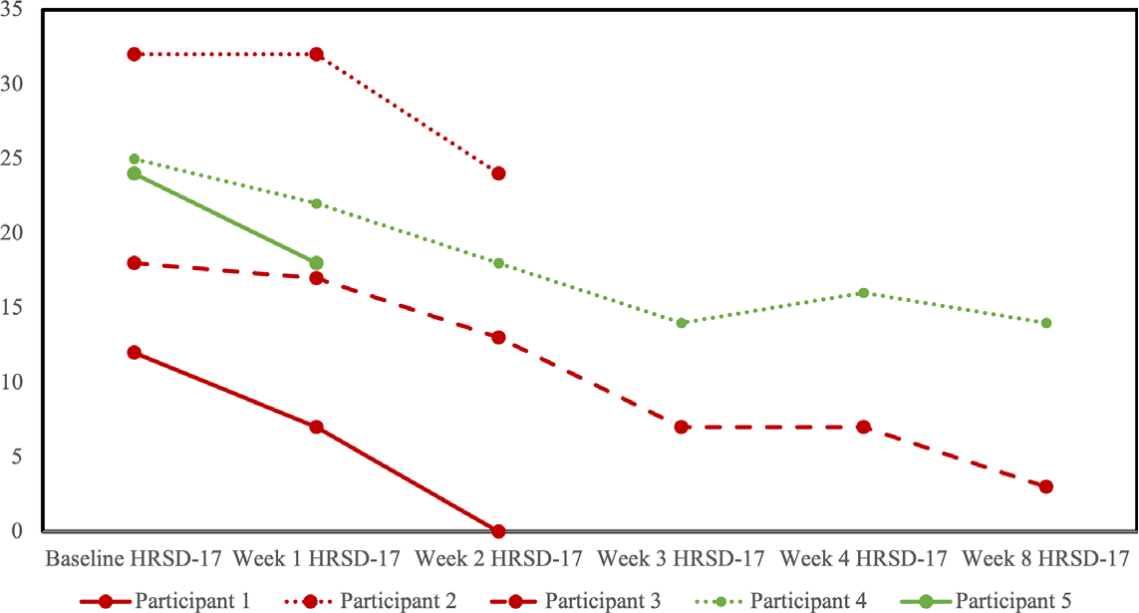
Profile plot of individual participants receiving sham (red) or active (green) repetitive transcranial magnetic stimulation (rTMS) and changes in the 17-item Hamilton Rating Scale for Depression (HRSD-17).

**Figure 4.**
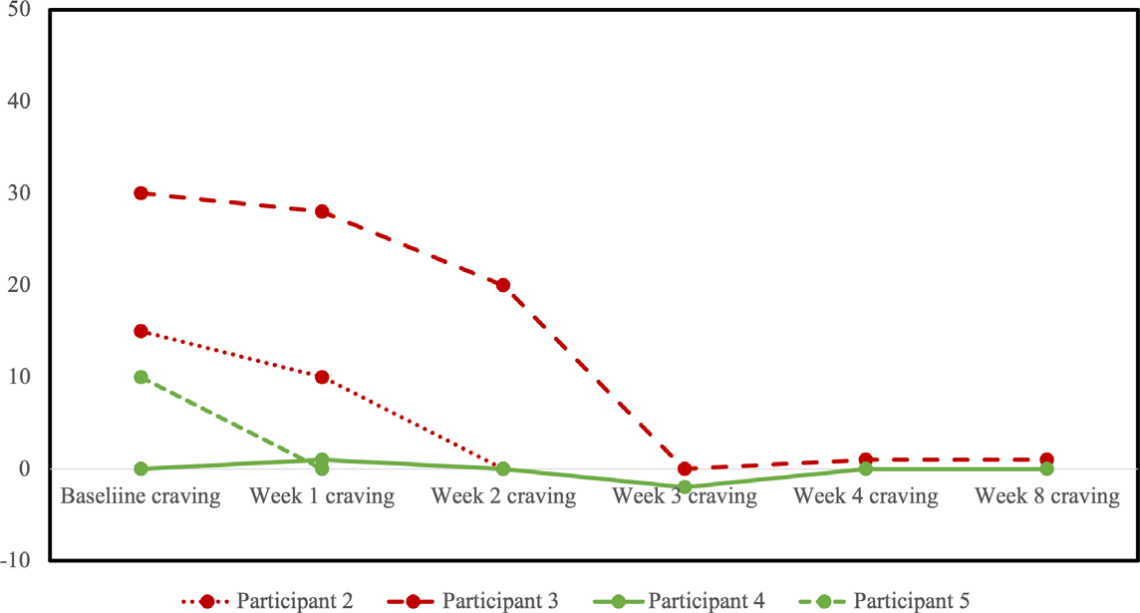
Profile plot of individual participants receiving sham (red) or active (green) repetitive transcranial magnetic stimulation (rTMS) and changes in cue-induced opioid craving. Craving scores shown represent the change in self reported craving on a visual analogue scale from 0 (no craving) to 100 (high craving) from pre- to post-exposure to a visual cue of opioid use.

HRSD-17 scores decreased for all participants from baseline to last observation (Figure 3). No participants reached the a priori definition of remission from the MDE, although two participants in the sham arm scored within the remitted range (≤7) on the last observation. Four participants completed at least one follow-up opioid craving assessment (Figure 4). The fifth participant could not tolerate the cue-induction procedure (viewing a video of injection drug use) and declined to participate in this part of the assessment in their subsequent follow-up visits. In three of four participants, there was a decrease from baseline to last observation in cue-induced opioid craving. The last participant completed the entire protocol but reported a zero or near zero ratings for all craving assessments throughout their entire study participation. No participants relapsed into opioid use for the duration of trial participation. One participant in the sham group (participant 2) was identified to have used cocaine and benzodiazepines on their urine drug screen in the week prior to dropping out. Five were daily tobacco smokers and two were regular cannabis users at baseline, and in the four participants with follow-up data, no changes in the pattern of use were detected throughout the trial.

### Safety

There were no serious adverse events for any participants. There were no adverse events appearing related to active rTMS. The most common adverse event was mild headache, reported in 2 in the sham group and 1 in the active group. The only other adverse event reported was insomnia.

## Discussion

We conducted a pilot feasibility study of a sham-controlled, randomized trial of bilateral rTMS to the DLPFC for the treatment of suicidality in patients with comorbid OUD and MDD. The protocol utilized multiple validated approaches including individual anatomical MRI targeting the DLPFC, TBS, which has been demonstrated to be a more efficient form of rTMS, a bilateral approach with cTBS to the right DLPFC and iTBS to the left DLPFC and a course of 20 daily treatment sessions. In terms of feasibility, the protocol was found to be safe and tolerable for participants with suicidal ideation and comorbid MDD and OUD. However, there were significant challenges in the recruitment and retention of participants, requiring termination of the trial without reaching recruitment targets. Although our trial generated interest among clinicians and patients over the two-year period, the majority of referred individuals could not be enrolled for a variety of reasons. Most commonly, participants were challenged to contact and schedule for the study procedures, likely owing to instability in their lives. Many presented with multimorbidity, unstable medical conditions, unstable housing, and financial situations, or complex personal and interpersonal situations. Similarly, for those who were able to enrol, one participant missed too many sessions due to deaths in the family and sickness, one could not afford transportation to treatment, and one had to withdraw due to a crisis involving intimate partner violence. During the course of the trial, several additional recruitment strategies were employed in an effort to increase recruitment, including adding compensation for time spent in all study visits, advertising to community addiction treatment providers and hospital partners, posting flyers for the public, sending letters to physicians and clinicians introducing the study, and advertising on classified advertisement websites. Several additional strategies to increase retention were also added, including adding compensation for each treatment visit attended, providing compensation for travel costs, leniency for missed treatment sessions in the event of physical sickness or extenuating personal circumstances, and changing the pre-treatment MRI to being an optional step that participants can decline. Despite these efforts, recruitment and retention rates did not increase enough to indicate the feasibility and justify the continuation of the trial.

OUD is associated with complexity and poor outcomes. Compared to the general population, OUD, opioid-related harms, and opioid overdoses have been associated with physical disability [27], childhood trauma [28], homelessness [29], being in a lower-income household [30], involvement in the criminal justice system [31], and more [32]. Furthermore, these risks for negative outcomes are especially increased with comorbid mental illness, such as the target population in this trial [8]. To develop the use of novel, neuromodulation therapies, including rTMS, for people with suicidality and OUD, this study highlights the need to first develop effective protocols that are adequately accessible for this complex population.

Despite the inconclusive findings, this study can be of interest to investigators planning to use neuromodulation for this combination of illness burdens. To the best of our knowledge, this is the first rTMS trial that has been conducted specifically to target suicidality in OUD, or indeed, any mental health comorbidity with OUD. This trial, conducted at a large urban centre in Canada, is the first published report of a RCT of rTMS in individuals with OUD outside of Asia. This is significant given the unique situation that North America is in, where the illicit opioid supply is now dominated by fentanyl and other synthetic opioids, resulting in an unprecedented opioid overdose crisis not seen elsewhere [1]. Indeed, previous trials of rTMS for individuals with OUD have been limited to individuals predominantly using heroin or morphine [23, 24, 33]. To our knowledge, this is the first report of rTMS to treat individuals with OUD with primarily fentanyl use. Given the association between opioid overdose deaths and suicide, it is of particular interest for research to be done on those who have a history of highly lethal substance use, such as fentanyl [34]. In this limited sample, a sham-controlled trial of bilateral TBS was shown to be safely conducted in patients with OUD in a North American context. Interestingly, most of the previous trials of rTMS for OUD have been done on inpatients admitted to a hospital or a rehabilitation facility [23, 24, 35], whereas this study was conducted on outpatients. It is possible that the limited ability to recruit and retain participants was related to this trial’s treatment setting and geographic location. Another reason for the challenges with recruitment was that many interested individuals were ineligible to participate due to the exclusion criteria, given the high prevalence of medical and psychiatric comorbidity in OUD. For example, many participants were excluded due to the presence of an unstable medical illness, taking an anticonvulsant or benzodiazepine medication, having a history of serious head injury, or having a significant history of seizures. These exclusion criteria were chosen for this trial because they are relatively common in rTMS trials for MDD and other psychiatric conditions and does not impede recruitment, but in the OUD population criteria may be too restrictive.

Overall, clinical outcomes for all participants in the trial were favourable, with the majority reporting improvement in suicidal ideation, depressive symptoms, and opioid cravings, but no clear differences between those who received sham or active rTMS. The small sample size prevented the undertaking of any meaningful statistical analysis, and even with a longer recruitment period, the study would likely remain underpowered to detect an effect. The limitations with sample size and recruitment are the most important limitations of the current study. Currently, there is a modest but promising evidence base on the effectiveness of rTMS for reducing suicidality in MDD without any other comorbidities [36, 37]. There has been some evidence that effects on reducing suicidality may be associated with a greater number of treatment sessions [37], bilateral rTMS [14, 38], and in combination with antidepressants [38]. The current literature suggests that rTMS has the potential to benefit patients in terms of both their comorbid MDD and OUD. The participants in this trial were selected for having an independent, primary, MDD, thus it is unclear whether these improvements were from the improvement of substance-induced mood symptoms and/or substance-related withdrawal symptoms. However, the reported study is the first specifically designed to recruit individuals with both conditions.

In conclusion, we conducted a pilot RCT of rTMS for suicidality in OUD with several novel aspects, including showing proof of concept of utilizing a state-of-the-art approach using an MRI-guided, bilateral TBS of a 20-daily treatment course, in patients with comorbid MDD and OUD. The major finding of infeasibility with respect to recruitment and retention highlighted the challenges of using rTMS to treat this highly complex comorbidity. However, given the large unmet need for novel treatments in this population and the potential efficacy of rTMS in reducing symptoms of suicidality, MDD, and opioid cravings, further research is warranted.

## Data Availability

Data may be available on requests aligning with REB permissions.
